# DNA recovery from wild chimpanzee tools

**DOI:** 10.1371/journal.pone.0189657

**Published:** 2018-01-03

**Authors:** Fiona A. Stewart, Alexander K. Piel, Lydia Luncz, Joanna Osborn, Yingying Li, Beatrice H. Hahn, Michael Haslam

**Affiliations:** 1 School of Natural Sciences and Psychology, Liverpool John Moores University, Liverpool, United Kingdom; 2 Department of Archaeology and Anthropology, University of Cambridge, Cambridge, United Kingdom; 3 Primate Archaeology Research Group, University of Oxford, Oxford, United Kingdom; 4 Departments of Medicine and Microbiology, University of Pennsylvania, Philadelphia, Pennsylvania, United States of America; Smithsonian Conservation Biology Institute, UNITED STATES

## Abstract

Most of our knowledge of wild chimpanzee behaviour stems from fewer than 10 long-term field sites. This bias limits studies to a potentially unrepresentative set of communities known to show great behavioural diversity on small geographic scales. Here, we introduce a new genetic approach to bridge the gap between behavioural material evidence in unhabituated chimpanzees and genetic advances in the field of primatology. The use of DNA analyses has revolutionised archaeological and primatological fields, whereby extraction of DNA from non-invasively collected samples allows researchers to reconstruct behaviour without ever directly observing individuals. We used commercially available forensic DNA kits to show that termite-fishing by wild chimpanzees (*Pan troglodytes schweinfurthii*) leaves behind detectable chimpanzee DNA evidence on tools. We then quantified the recovered DNA, compared the yield to that from faecal samples, and performed an initial assessment of mitochondrial and microsatellite markers to identify individuals. From 49 termite-fishing tools from the Issa Valley research site in western Tanzania, we recovered an average of 52 pg/μl chimpanzee DNA, compared to 376.2 pg/μl in faecal DNA extracts. Mitochondrial DNA haplotypes could be assigned to 41 of 49 tools (84%). Twenty-six tool DNA extracts yielded >25 pg/μl DNA and were selected for microsatellite analyses; genotypes were determined with confidence for 18 tools. These tools were used by a minimum of 11 individuals across the study period and termite mounds. These results demonstrate the utility of bio-molecular techniques and a primate archaeology approach in non-invasive monitoring and behavioural reconstruction of unhabituated primate populations.

## Introduction

Genetic studies that target the evolutionary history of hominin individuals, groups and species have revolutionised human archaeology over the past decade. These studies have revealed the existence of previously unknown taxa [1] and demonstrated both genetic diversity [2] and migration patterns [3] that would otherwise remain unknown. As this work has progressed, data from genomic and more recently proteomic approaches have been tied back to the tools that accompany the skeletal archaeological record [4]. These new approaches provide a richer context for understanding the suite of behavioural changes that took place in the Late Pleistocene hominin lineage.

The general absence of skeletal remains from past populations of African great apes [5] currently precludes the use of the same molecular approach to reconstruct ancient ape evolution. However, a modern-day analogy presents itself in the numerous great ape populations that remain unhabituated to human presence, and therefore whose behaviour goes undescribed and more broadly, undetected. For wild chimpanzees (*Pan troglodytes*), for example, of over about 150,000 remaining individuals, our cumulative dataset is comprised from fewer than ten medium or long-term sites [6]. This bias has limited studies of wild chimpanzees to an unrepresentative subset of communities that are known to show great behavioural diversity on small geographic scales.

Three different approaches have been used to increase information on unhabituated wild chimpanzee populations through non-invasive monitoring. First, genetic data from living apes have allowed reconstruction of past population sizes and interbreeding events leading to the current species [7–10]. Non-invasive genetic sampling methods also have been developed to provide information on chimpanzee populations where direct observations of behaviour are impossible or very rare. These methods have led to a better understanding of ranging patterns, population estimates [11,12], and kin relationships [13–15].

A second approach involves the recording of material evidence, such as abandoned tools or information on nests, from chimpanzee home ranges. This technique has proven useful for assessing the behavioural repertoire of wild chimpanzee populations [16–23]. However, in both of these approaches the behaviour of individual group members cannot typically be reconstructed, and potential diversity of behaviours between individuals is therefore lost. Where such behaviours involve tool use, we cannot, for example, assess tool selection or modification practices at an individual level, leaving questions of social influence and traditions unanswerable [24].

A third approach of camera trapping at potential tool use sites does allow for learning about tool use behaviours at the individual level in unhabituated communities [25]. However, the field of view of the camera can miss individuals that are present, and individuals are not always identifiable depending on the images captured [26].

**Fig 1:**
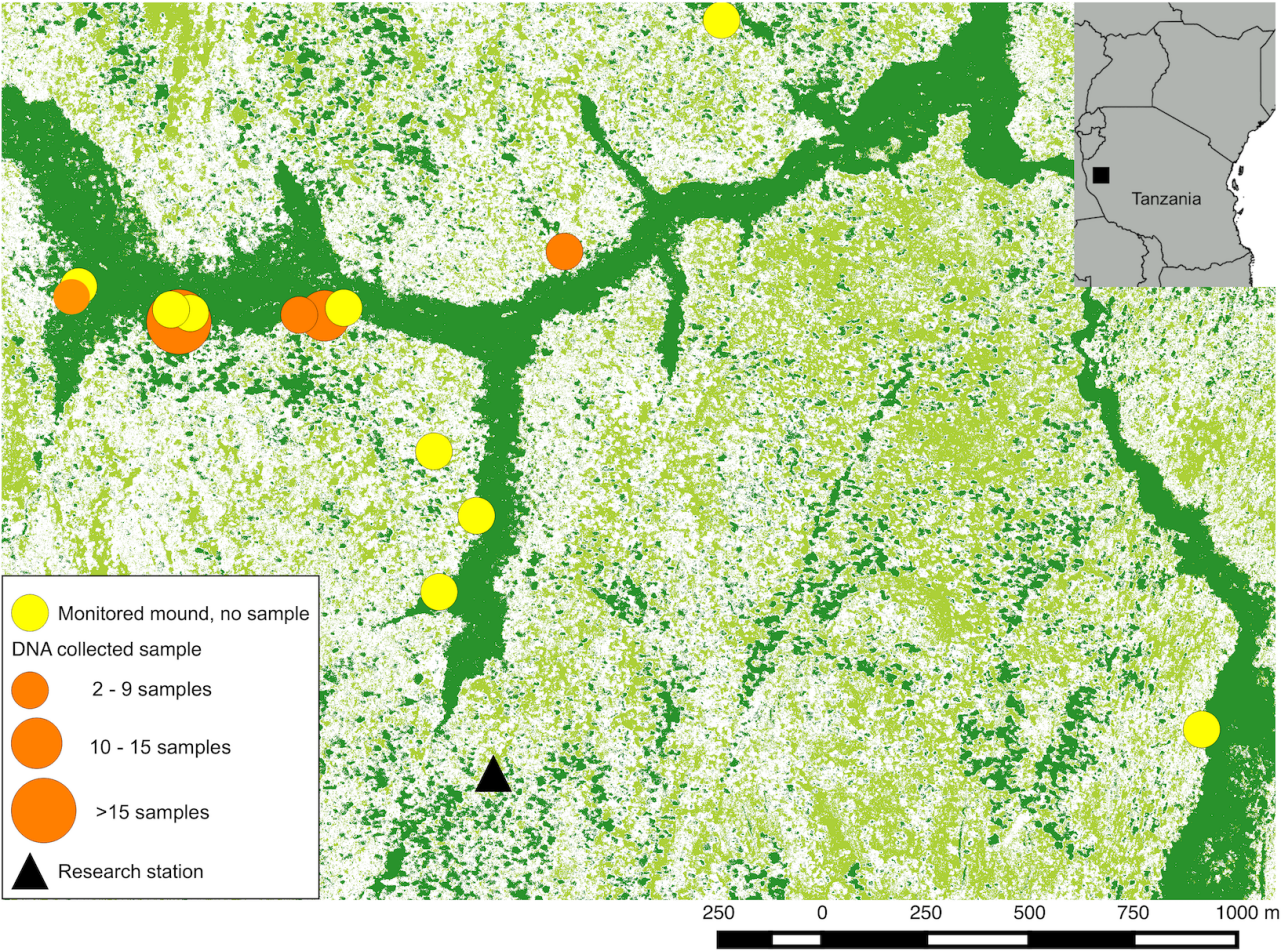
Map with sampled sites, and number of tools recovered from each site. Vegetation classification (evergreen forest—dark green; deciduous woodland–light green; grassland–white) by Lilian Pintea, Jane Goodall Institute (JGI).

Here, we introduce a new method that bridges the gap between behaviour and material evidence in unhabituated chimpanzees and the application of genetic advances to the field of primatology. Our analysis focuses on genetic evidence recovered from wild Eastern chimpanzee (*P*. *t*. *schweinfurthii)* termite-fishing tools. As chimpanzees mouth these plant tools to remove termites, epithelial cells are transferred to the tools in a manner that resembles mouth swabs used in human genetic sampling. The chimpanzees in this study live in the mosaic woodlands of the Issa Valley in western Tanzania (Fig 1), and direct observation of their behaviour is less reliable than at longer-term sites where chimpanzees are well habituated to the presence of human observers and typically occupy smaller, forest dominated home ranges [27]. In this study, we aimed to (i) identify whether termite fishing leaves behind detectable chimpanzee DNA evidence, (ii) quantify the DNA recovered and compare it to other recovery techniques (e.g., faecal sampling), and (iii) perform an initial assessment of the feasibility of using mitochondrial and microsatellite markers to determine the minimum number of tool users in our sample. The results help establish a new approach combining molecular and archaeological methods to reconstructions of primate behaviour.

## Methods

### Study site

Samples were collected at the Issa Valley research site in western Tanzania (S 5.50°, E 30.56°) between October 2014 and January 2015. Permission to conduct research at Issa and export samples was granted by the Tanzanian Wildlife Research Institute (TAWIRI) and Commission for Science and Technology (COSTECH). Samples were imported to the UK under (DEFRA) permit TARP/2014/236. Issa lies approximately 90 km east of Lake Tanganyika, within the Greater Mahale Ecosystem (GME). The GME is characterized by broad valleys separated by steep mountains and flat plateaus ranging from 900 to 2100 m elevation. The vegetation is dominated by miombo woodland (*Brachystegia* and *Julbernardia*, Fabaceae), although it also includes swamp and grassland, as well as evergreen gallery and thicket riverine forests. There are two distinct seasons: wet (October–April) and dry (May–September), with dry months defined as having <100 mm of rainfall. Termite fishing (*Macrotermes spp*.) occurs predominantly during the wet season [27]. Rainfall averages 1250 mm per annum [28], and temperatures range from 11 to 35°C [27]. Chimpanzees in Issa are partially habituated, and research focuses on an 85 km^2^ study area. Preliminary genetic analyses identified at least 67 individuals, including 31 females and 27 males (and 9 individuals that could not be sexed definitively), within the Issa community [29]. As of December 2016, eleven chimpanzees were individually recognizable to researchers.

### Sample collection

Thirty termite mounds were monitored three to four times weekly for chimpanzee activity. All new termite-fishing probes that were abandoned in situ where they had been inserted into a mound were collected for DNA analyses. Each tool was collected using a new pair of gloves. A 5 cm segment of the end of the tool that was inserted for fishing was cut using scissors sterilized with bleach and ethanol between each use and preserved in 5 ml of RNA later (Life Technologies). Faecal samples were collected from within the Issa chimpanzee study area between 2009 and 2012. They were collected from trails when tracking chimpanzee parties, or from beneath chimpanzee night nests, and preserved in an equal volume of RNAlater.

All samples were kept frozen at -18°C on site in a solar powered DC freezer (Model number ARB, 47L), before shipment at room temperature. Termite tools were shipped to the University of Cambridge in January 2015 and stored at -20°C, whilst chimpanzee faeces were shipped several times yearly to the University of Pennsylvania and stored at -80°C.

### DNA extraction and quantification

DNA was extracted from the chimpanzee tools using two commercially available forensic DNA kits: the QIAamp® DNA Investigator Kit for use with the Qiacube system and the DNA IQ™ Casework Pro Kit for Maxwell 16®. Extractions took place inside a UV sterilised laminar flow hood and gloves were changed between samples to prevent human contamination. All consumable plastics used were single use only and scissors/ forceps were sterilised in an auto-clave in order to prevent cross-contamination between chimpanzee samples. In each case, DNA was extracted from a 1 cm section of each tool following the manufacturer’s protocol for trace DNA extraction from a solid substrate, and approximately 60 μl and 50 μl, respectively, of DNA was eluted. We alternated the extraction method that was used for the first (tip) or second 1cm section of tool. DNA extraction from faeces followed previously described methods [30,31]. Approximately 0.7 ml RNAlater preserved stool was used in each extraction.

Real-time quantitative PCR (qPCR) followed methods described by Morin et al. [32] to amplify an 81bp portion of the chimpanzee c-myc gene of all samples. qPCR amplification of chimpanzee DNA from termite tools and faeces was performed in a 7500 RT PCR system (Applied biosystems) and triplicate sets of size standards of known DNA concentration and negative controls were included with each set of samples prepared with a single qPCR reaction mix. Analyses were conducted in DataAssist™ Software and were checked using standard curves and calculations in Microsoft Excel. Chimpanzee DNA quantification was performed for each DNA extract (QIAamp and DNA IQ) in quadruplicate to calculate an average quantity of chimpanzee DNA per sample.

### Mitochondrial genotyping

A 498 bp fragment of the mitochondrial (mt) D-loop region was amplified from termite tool DNA extracts using primers L15996-M13RpUC[33] and H16498-M13F[34]. PCR amplification was performed in a total volume of 25 μl consisting of 3 μl template, 1x PCR buffer, 3mM MgCl_2_, 200 μM of each dNTP, 1μg BSA, 1 μM each primer, and 1.25 U Bioline Taq polymerase. Amplification conditions were: initial denaturation at 94°C for 1 min, 40 cycles of 20s at 94°C, 30s at 55°C, 1 min at 72°C, and a final extension at 72°C for 10 min. Where amplification was unsuccessful a nested protocol was used by first amplifying the whole D-loop using outside primers L15926 [35] and CEH5 [36] followed by inside amplification as described above, excluding BSA. Products were separated on a 1% agarose gel (100 V, 15 min) and visualized using Et Br and UV light. PCR products were sent for sequencing at Macrogen (Korea). Independent PCR products of each tool extraction were sequenced in both directions and sequences were aligned using the Clustal W function in MEGA 5.1. Termite tool derived sequences were subjected to phylogenetic analyses to identify distinct mtDNA haplotypes, all of which matched previously identified haplotypes from this same region [29].

### Microsatellite genotyping

Only samples with more than 25 pg/μl were selected for microsatellite genotyping, as previous research has shown that reactions containing <100pg/rxn require seven replicates to determine genotypes with high confidence, whilst 101-200pg/rxn requires four and >201pg/rxn requires only two replicates for similar confidence [32]. Two microsatellite loci known to be variable in this population (d5s1457 and d2s1326) [12] and one locus known to be variable in chimpanzees in general (d1s550) [37] were amplified from termite-fishing tool DNA extracts in a single-step multiplex PCR reaction.

PCR amplification was carried out in a total volume of 9 μl, consisting of up to 4 μl of template, 4.5 μl of 2X Qiagen multiplex mastermix, 200nM of each primer, and 60ng BSA. Amplification conditions were: initial denaturation at 95°C for 15 min, 40 cycles of 95°C for 30s, 60°C for 90s, 72°C for 60s, and a final extension of 30 min at 72°C. The 5’ end of the forward primer was fluorescently labeled, and products were separated using capillary electrophoresis (ABI 3730xl DNA analyser). Alleles were then sized relative to a size standard (HD400 labeled ROX) using Geneious 7.1.9 with microsatellite plugin. A modified multiple-tubes approach was adopted based on the quantity of DNA in each extract. Following Morin et al. [32], two replicates of two alleles were required to call a heterozygous locus, four replicates were required to call a homozygous locus if there was 100-200pg/rxn and two replicates if there was >200pg/rxn.

### Analyses

We estimated allelic dropout following Gushanski et al. [38], by dividing the total number of allelic dropouts observed by the total number of successful heterozygous reactions, across all loci. Ideally, a larger number of microsatellite loci would be used to determine individual identity with more certainty; however, three loci are sufficient for testing the feasibility of microsatellite genotyping of termite-fishing tool DNA extracts. In order to determine a minimum number of individuals represented from the tool samples and any potential matching genotypes we used CERVUS 3.0 [39] to perform an identity analysis and assess the probability of full siblings or unrelated individuals having an identical genotype (pIDsib and pID).

## Results

### Sample collection

Twelve of the thirty monitored termite mounds were actively fished during the monitoring period, and tools were collected for DNA analysis from six mounds. We collected 49 tools on 11 occasions between November 2014 and January 2015 (Table 1; Fig 1). Between one and six tools were collected from each fishing episode. Five out of the six mounds were fished on more than one occasion. All tools were made of stripped bark and averaged 410±230 mm in length and 5±2.6 mm in width, conforming to initial descriptions of termite tool characteristics at Issa [27]. Of 450 faecal samples collected from Issa between 2009 and 2012, 313 were subjected to DNA extraction and quantification.

**Table 1:** Genetic identity (ID), genotype and mtDNA haplotype for each tool.

Date	Tool Number	Termite Mound	mtDNA haplotype*	Individual genetic ID	d5s1457	d1s550	d2s1326
17/10/14	1	4503	GM7				
17/10/14	2	4503	GM7				
01/11/14	1	4862	GM7	1	110/114	154/166	210/218
01/11/14	2	4862	UG59	2	114/114	150/154	206/218
01/11/14	1	5298	GM7	3	110/114	154/154	206/210
01/11/14	2	5298	GM7				
01/11/14	3	5298	GM7				
07/11/14	1	5298	GM7	4	102/114	150/150	218/218
07/11/14	2	5298	GM7	5	110/114	166/166	206/206
07/11/14	3	5298	GM7	6	102/114	150/150	210/218
07/11/14	4	5298	GM7				
09/11/14	2	5298	GM7	6	102/114	150/150	210/218
09/11/14	3	5298	MH37	7?	114/122	150/150	210/218
09/11/14	4	5298	MH37	7	114/122	150/154	210/218
09/11/14	5	5298	MH37	8	106/114	150/154	214/218
09/11/14	6	5298	UG59	9	114/114	150/170	210/210
11/11/14	1	5298	UG59	10	106/114	150/154	206/218
11/11/14	2	5298	UG59	11	106/126	150/154	206/218
11/11/14	3	5298	UG59	2	114/114	150/154	206/218
19/11/14	1	4502	GM7				
19/11/14	1	5298	GM7	12	102/114	170/170	206/218
21/11/14	1	4030	GM7	6	102/114	150/150	210/218
21/11/14	2	4030	GM7	6	102/114	150/150	210/218
30/11/14	1	4030	UG59	13	106/126	154/154	206/218
30/11/14	3	4030	UG59				
30/11/14	1	4502	UG59				
17/12/14	1	4769	UG59				
17/12/14	2	4769	UG59	2	114/114	150/154	206/218
17/12/14	4	4769	UG59				
17/12/14	1	5298	GM7				
19/12/14	1	5298	UG59	14?	102/114	154/166	186/218
08/01/15	1	4502	UG59				
08/01/15	3	4502	GM7				
08/01/15	5	4502	UG59	2?	114/114	150/154	206/218
08/01/15	6	4502	UG59				
08/01/15	1	4769	UG59	15	102/114	150/150	210/218
08/01/15	2	4769	GM7	6	102/114	150/150	210/218
08/01/15	3	4769	GM7	6	102/114	150/150	210/218
08/01/15	1	5298	UG59				
08/01/15	2	5298	GM7	16	102/114	154/154	186/218
08/01/15	3	5298	UG59	14	102/114	154/166	186/218

* Genbank accession numbers for haplotypes GM7-DQ370321, UG59-JN091703, MH37-EU527467.

Shaded cells contain unconfirmed genotypes (with fewer than necessary replicates per DNA quantity, see methods) and blank cells represent no data.

? These IDs are possible matches, but genotypes remain unconfirmed.

### Chimpanzee DNA quantification

**Fig 2:**
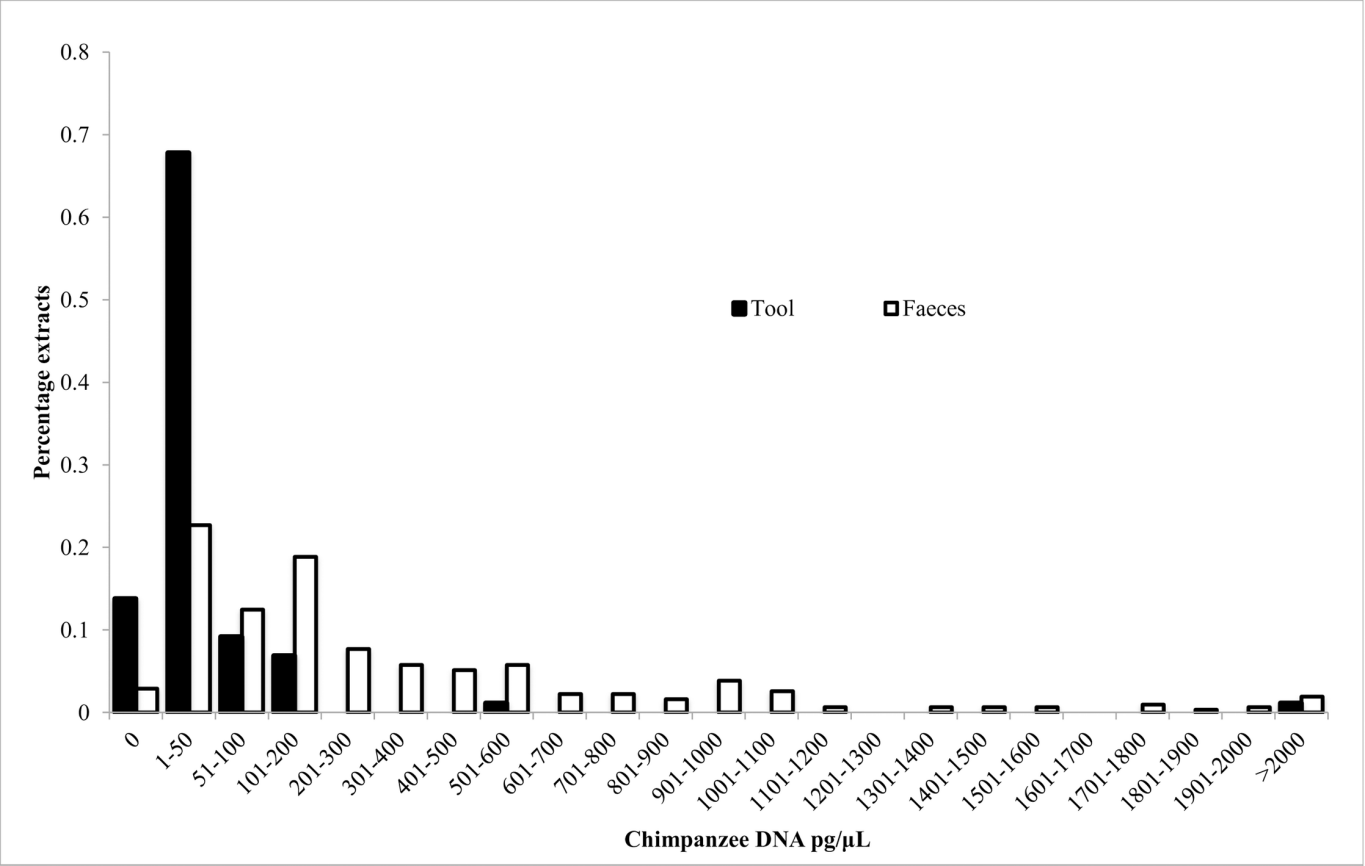
Histogram comparing chimpanzee DNA concentration of termite-fishing tool and faeces DNA extracts.

The average chimpanzee DNA concentration found in tool DNA extracts was 52 pg/μl (range 0–2080; Fig 2). There was no difference in chimpanzee DNA quantity between DNA extracted using the QIAamp® DNA Investigator kit or the DNA IQ™ Casework Pro Kit (Wilcoxon’s matched pairs V = 704, p = 0.14). There was no difference in chimpanzee DNA quantity between first and second extracts of each tool (Wilcoxon’s rank sum test; W = 793.5, p = 0.24). Extracts with <5 pg/μl chimpanzee DNA were considered unusable for microsatellite analyses [32] and 37% of all extracts had <5 pg/μl chimpanzee DNA (15 or 31% of QIAamp and 21 or 43% of IQ™ Casework extractions).

The extractions with <5pg/μl chimpanzee DNA did not result from the same tools. For example, nine samples yielding unusable extracts with the QIAamp® Kit (mean 2.2 pg/μl), yielded usable extracts with the DNA IQ™ Kit (mean 31 pg/μl) and three samples yielding unusable extracts with the DNA IQ™ Kit (mean 2.9 pg/μl), yielded usable extracts with the QIAamp® Kit (mean 11.8 pg/μl). In comparison, the average chimpanzee DNA concentration found in faeces extracts was 376.2 pg/μl (range 0–4867; Fig 1) and only 16 samples, or 5% of extractions, yielded <5 pg/μl of chimpanzee DNA.

### Mitochondrial DNA

PCR amplification success rate for HV1 mitochondrial DNA from tool samples was 86% (197 of 228 PCRs). There was no difference in amplification success rate between DNA extracted using the QIAamp® DNA Investigator kit (88% or 88 of 104 PCRs) or the DNA IQ™ Casework Pro Kit (85% or 109 of 124 PCRs). Extracts that yielded mtDNA HV1 sequences had significantly more DNA (mean 66.7 pg/μL, range 0–2080 pg/μl) than those that did not (mean 2.8, 0–20.6 pg/μl; Wilcoxon’s rank sum test, W = 209, p<0.0001). mtDNA haplotypes could be assigned to 41 of 49 tools (Table 1). All tools had one of three haplotypes known for this community of chimpanzees [29] and the wider GME region [40].

### Microsatellite genotyping

**Fig 3:**
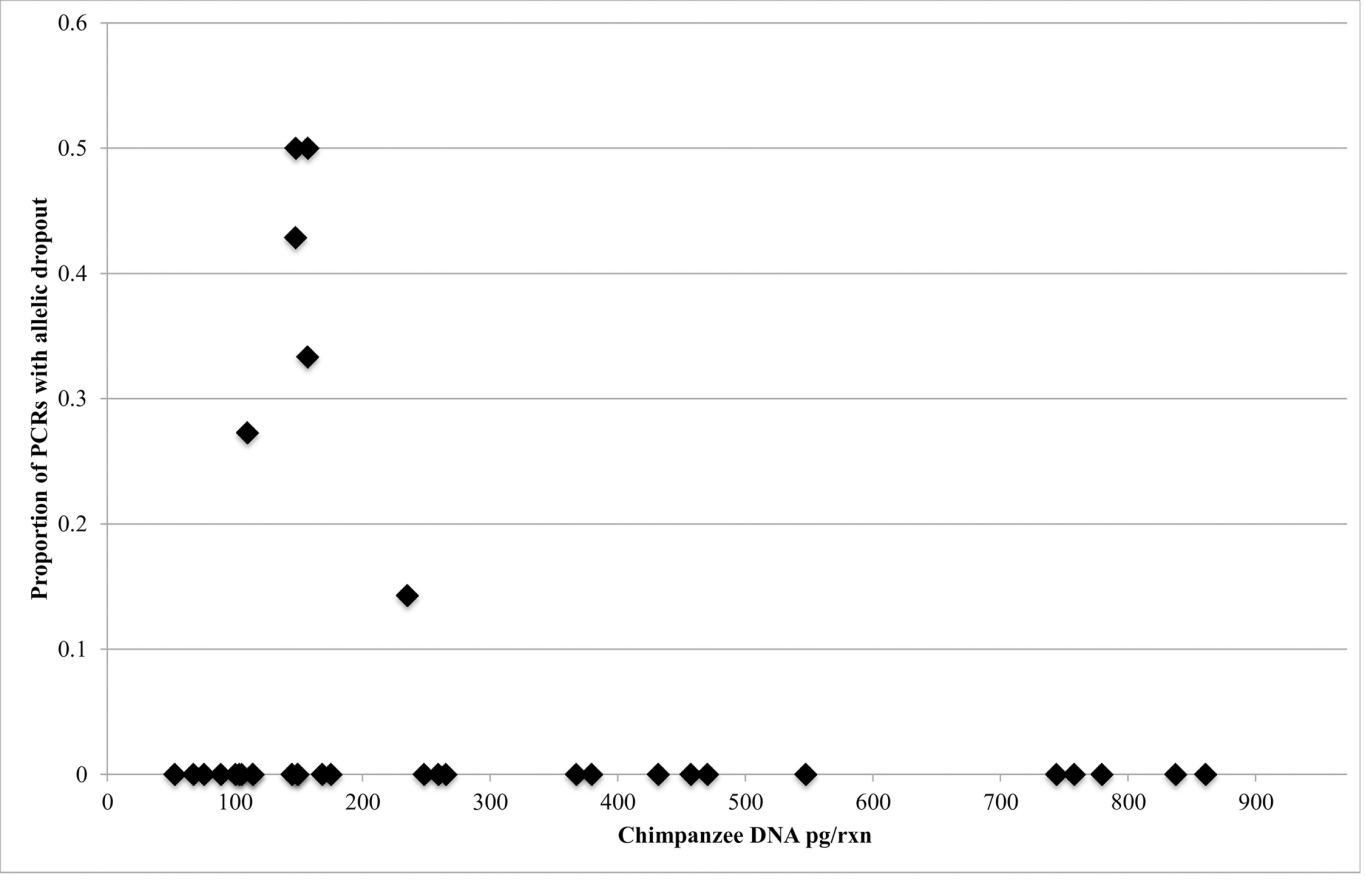
Proportion of PCRs with allelic dropout in relation to the concentration of termite-fishing tool DNA extract.

Twenty-six tool DNA extracts yielded >25 pg/μl chimpanzee DNA and were selected for microsatellite analyses. All loci amplified in at least one replicate per extract. Results were attained for between two and seven replicates for each tool, which allowed genotypes to be determined with confidence for 18 of 26 tools (Table 1). The extent of dropout across all heterozygous loci was 8%, and only one case (0.06%) of irreproducible sporadic alleles was observed. Dropout was not observed in samples with more than 235pg/rxn (Fig 3).

Identity analysis in CERVUS was used to find matching genotypes within the 18 confirmed genotypes. These tools represent a minimum of 11 different individuals; two genotypes occurred more than once, representing three and six tool samples used by two different individuals (Table 1; IDs 2 and 6 respectively). If unconfirmed genotypes are included in the analysis, the 26 tools were used by a minimum of 16 individuals, and four tools could potentially have been used by a further two different individuals (Table 1; IDs 7 and 14). However, the theoretical probability of two full siblings or two unrelated individuals sharing the same genotype at three loci was high (pIDsib = 0.15, pID = 0.021) given the limited number of loci genotyped.

Tools re-used by the two confirmed individuals were similar in length, but may differ in width; ID 2 tools averaged 400±260 mm in length and 6.3±3.2 mm in width, whilst ID 6 tools averaged 430±150 mm in length and 3.8±1.2 mm in width.

## Discussion

This study presents the first successful extraction, mitochondrial DNA amplification, and microsatellite genotyping of DNA from material artefacts left behind by wild chimpanzees. These results demonstrate the utility of molecular techniques and a primate archaeology approach [41] in non-invasive monitoring and behavioural reconstruction of unhabituated primate populations. Our findings are the first step in linking non-invasive genetic techniques, traditionally used with extant non-human primates, to the study of material culture remains left from hominin landscape use. Our results validate a genetic approach to the recovery of otherwise unobtainable behavioural information directly related to tool use by wild animals.

Our monitoring program identified and recovered chimpanzee termite-fishing tools from multiple mounds, and on multiple occasions from single mounds. Genetic identification of tool users opens up opportunities to record unobserved chimpanzee behaviour, including inter-site ranging and regularity of site visits. They also allow us to begin cataloguing individual chimpanzee preferences for tool size, shape and material, and to do so over any time period during the life of the animal. For example, the tools of two individuals identified in this study show a difference in the width of tools manufactured. A longitudinal study with a larger sample size would determine whether this difference is significant.

The mtDNA analysis placed all recovered genetic material into known haplotypes of the study community [26] and region [40]. This result is unsurprising; however we anticipate that if future tool collection expands into less well-studied parts of the GME, we will begin to see additional haplotypes. Ultimately, we expect that we will be able to assess whether there is a potential long-lasting maternal influence on tool forms, by comparing data on tool material selection and manufacture between the different mitochondrial lineages, akin to what has recently been reported for hand-clasping in Ugandan chimpanzees [42]. Our initial hypothesis would be that the patrilocal nature of chimpanzee society, coupled to the sustained cultural variation seen between neighbouring groups [43], would act to diffuse any matriline-specific technological attributes, but at this point that remains an open question.

Tool transfers do occur between wild chimpanzees; Mothers have been observed to share tools with their offspring [25], adult tool sharing has been observed in a similarly dry site [44], and chimpanzees have been seen to sniff and inspect tools found on mounds. Despite this, we found no evidence of cross-contamination in our study. Such tool transfers might not occur at Issa, or might occur at a rate too low to have been detected here in a small sample of tools. Cross-contamination by multiple chimpanzees using the same tool could therefore be an issue to be addressed in future research using this method.

Our microsatellite analysis is preliminary, but demonstrates feasibility of identifying tool users directly from the tool. If we assume that genotypes matching at three loci represent the same individual, we found that one individual (ID 6 in Table 1) may have used up to six of the tools recovered during this study. These tools were collected from three different termite mounds, across a period of two months. Archaeologically, this ability to track an individual’s use of specific tools over that time is the equivalent of attempting to track the products of an individual stone knapper at a Palaeolithic human site [e.g., 45], but with the added detail and linkages provided by the genetic data. We anticipate that the routine and long-term application of our methods at a single site would reveal the links between social and genetic influences on tool selection and modification at a level that is currently unobtainable. We expect that the extension of this work to habituated wild chimpanzee sites would yield a similar increase in knowledge, as even these sites cannot directly observe and collect all evidence of tool use all the time [46]. Genetic analysis of tools would complement well studies of tool use in habituated and unhabituated apes using camera traps (e.g. [25]), as observation and video of tool use provides information about age classes not obtainable from genetics.

The chimpanzee DNA quantity in our tool extracts (52 pg/μl) was lower than that found in commonly used DNA extracts from faeces, but was significantly higher than that of shed hair samples (4.4pg/μl; Morin et al., 2001). Likewise, although a large proportion of tool DNA extractions (37%) contained <5pg/μl chimpanzee DNA, this is lower than 79% of extractions of shed hair found by Morin et al [32] to contain 0pg/μl chimpanzee DNA. Hair extractions typically use only a single hair follicle, in order to be certain that only one individual is sampled. Differences in chimpanzee DNA quantity may be influenced by the amount of starting material, or due to greater sensitivity of forensic techniques, or tools may actually be a better source of chimpanzee DNA than shed hairs. RNAlater might also improve the yield of DNA extractions, as we found a much higher concentration of DNA in extractions from faeces than Morin et al. [32] (192 pg/μl), who preserved faeces in silica gel and used 100mg dried faeces for DNA extraction. The Issa environment is also much drier than that of the Tai forest, where Morin et al. [32] collected samples. Such a difference in environment, or possible differences in the time since deposition to collection of samples, could influence DNA yield. Tools are also beneficial as a DNA source as they provide more than one opportunity to extract DNA to potentially improve yield, similar to faeces, whereas hair samples allow only a single extraction. The two forensic kits that we employed were found to be equally reliable, with no difference in the DNA quantity extracted nor in ease of implementation. Both kits also have a very similar cost per microliter of extracted DNA of around 3.5 μl/£1.

A logical next step for this work would be to identify non-chimpanzee activities that similarly may result in individual DNA preservation on tools. Human ancestors, for example, used a wide variety of plant and stone tools in the past, and while the perishable nature of much material culture necessarily places restrictions on that search, DNA recovery from human stone tools is possible [47]. Similarly, stone tools have been recovered that were used by past groups of capuchin monkeys (*Sapajus libidinosus*) and long-tailed macaques (*Macaca fascicularis aurea*) to open encased foods [48,49], and populations of orangutans (*Pongo pygmaeus*) are habitual tool users that extensively orally manipulate plant tools [50]. We recognise that the technique introduced here is likely to be more effective for some tool types and tasks than others, but we also believe that the ultimate benefits of successfully linking genetic and technological records are sufficient to warrant the continued exploration of its potential.
